# Predictors and long-term impact of sustained complete renal response in lupus nephritis patients over a median 10-year follow-up

**DOI:** 10.1093/rheumatology/keaf677

**Published:** 2025-12-13

**Authors:** Ioannis E Michelakis, Eleni Kapsia, John N Boletis, Smaragdi Marinaki, Petros P Sfikakis, Maria G Tektonidou

**Affiliations:** First Department of Propaedeutic Internal Medicine, Joint Academic Rheumatology Program, Laiko Hospital, Medical School, National and Kapodistrian University of Athens, Athens, Greece; Department of Nephrology and Renal Transplantation, Laiko Hospital, Medical School, National and Kapodistrian University of Athens, Athens, Greece; Department of Nephrology and Renal Transplantation, Laiko Hospital, Medical School, National and Kapodistrian University of Athens, Athens, Greece; Department of Nephrology and Renal Transplantation, Laiko Hospital, Medical School, National and Kapodistrian University of Athens, Athens, Greece; First Department of Propaedeutic Internal Medicine, Joint Academic Rheumatology Program, Laiko Hospital, Medical School, National and Kapodistrian University of Athens, Athens, Greece; First Department of Propaedeutic Internal Medicine, Joint Academic Rheumatology Program, Laiko Hospital, Medical School, National and Kapodistrian University of Athens, Athens, Greece

**Keywords:** systemic lupus erythematosus, lupus nephritis, sustained renal response, kidney function decline, damage accrual

## Abstract

**Objectives:**

Complete renal response (CRR) is a primary goal in lupus nephritis (LN) management. We examined the prevalence and predictors of sustained CRR (sCRR) and long-term outcomes.

**Methods:**

We included 142 inception cohort patients with biopsy-proven LN from two academic centres. We assessed the prevalence of sCRR achievement for ≥12 months and the impact of sCRR duration on renal flares, severe kidney function decline (≥30% eGFR decline compared with baseline), a composite end-stage kidney disease (ESKD) or death outcome, and disease damage. We analysed data over a median 121-month follow-up, using linear, logistic and Cox regression models.

**Results:**

A total of 83% of patients achieved sCRR for ≥12 months, 56.3% for ≥5 years and 20.4% for ≥10 years. Persistent hydroxychloroquine use (adjusted HR: 1.86, *P* = 0.004), non-nephrotic baseline proteinuria (adjusted HR: 1.71, *P* = 0.016) and class III *vs* class IV LN (HR: 1.89, *P *= 0.018) were associated with earlier sCRR achievement. The 5- and 10-year post-sCRR risks for renal flares decreased for every additional year on CRR. sCRR duration rather than its mere achievement reduced the risk of ≥30% eGFR decline (adjusted OR: 0.81/year, *P *= 0.015) and composite ESKD/death (adjusted HR: 0.75/year, *P *= 0.001). sCRR ≥ 12 months protected against damage accrual (adjusted β-coef=−1.17, *P* < 0.001). Among those with ≥100-month follow-up, sCRR ≥ 4 years protected against severe kidney function decline (adjusted OR: 0.10, *P* = 0.005), ESKD/death (adjusted HR: 0.11, *P *= 0.043) and damage accrual (adjusted β-coef=–0.81, *P* = 0.012).

**Conclusion:**

Persistent hydroxychloroquine, non-nephrotic baseline proteinuria and class III *vs* IV are associated with earlier sCRR. sCRR ≥4 years protects against ≥30% eGFR decline, composite ESKD/death and damage.

Rheumatology key messages

- Persistent hydroxychloroquine use is associated with shorter time to achieve sustained complete renal response (sCRR).
- Earlier sCRR achievement and sCRR duration, rather than simply achieving complete response, better predict long-term outcomes.
- sCRR≥4 years protects against ≥30% eGFR decline, ESKD/deaths and damage accrual.

## Introduction

Lupus nephritis (LN) is the most common and severe manifestation of systemic lupus erythematosus (SLE), affecting 25–50% of patients and significantly increasing the risk of morbidity and mortality [1–5]. Despite advances in disease management over the past decades, 10–30% of patients with LN may develop end-stage kidney disease (ESKD) [6].

Achieving complete renal response (CRR) is a key treatment goal in the management of LN, as it is associated with improved renal and patient outcomes [7–9]. Renal flares, however, are common throughout the disease course, occurring in 30–40% of patients and leading to a gradual loss of nephrons and progressive decline of kidney function [2, 7, 8]. Furthermore, patients with frequent renal flares are at a higher risk of experiencing steroid-related side effects [10]. In this context, maintaining renal response emerges as a more important treatment goal than simply achieving it. Previous studies examining sustained remission in LN, highlighted its benefits on kidney disease progression and damage accrual. However, definitions of sustained remission have varied across studies [11–14], limiting comparability and clinical adoption. CRR, as defined by the EULAR/ERA-EDTA guidelines and KDIGO recommendations [15–17], represents the most widely accepted and clinically relevant treatment target. The minimum duration of CRR required for long-term renal protection remains uncertain, and it is also unclear whether prolonged CRR can be achieved and maintained with reduced exposure to immunosuppressive and glucocorticoid therapy.

In this study, we aimed to assess: (i) the prevalence and duration of CRR; (ii) predictors of the time to achieve sustained CRR (sCRR); (iii) the impact of sCRR on flares, severe kidney function decline, ESKD/death composite outcome, damage accrual; and (iv) the minimum CRR duration required to prevent adverse long-term outcomes.

## Methods

### Study population

We examined 142 inception cohort LN patients diagnosed between 1992 and 2021, and followed up for a minimum of 3 years at two joint academic centres (Rheumatology and Nephrology Units, Laiko Hospital). All patients had biopsy-proven LN (classes III, IV, V, III/IV+V) according to the 2003 ISN/RPS LN classification system [18] and met the 2019 SLE classification criteria [19]. The study was approved by the Institutional Review board at Laiko Hospital (protocol number: 14697/08-11-2022). Informed consent was not required (retrospective study, use of anonymized data).

### Data collection

Clinical/laboratory, histological and treatment data for each patient were recorded at the time of LN diagnosis (baseline), at 3, 6, 9, 12, 18, 24 months and yearly thereafter until the end of follow-up (EFU). The collected data included: demographics, time from SLE diagnosis until LN diagnosis, anti-ds DNA antibodies, C3 and C4 levels, estimated glomerular filtration rate (eGFR, based on the CKD-EPI formula), urine protein excretion (assessed by 24-h urine collections), urine sediment, kidney biopsy findings [LN class, activity index, chronicity index, crescents, interstitial fibrosis/tubular atrophy (IF/TA) and glomerulosclerosis]. All biopsies contained ≥10 glomeruli and those performed before 2003 were reassessed based on the ISN/RPS 2003 classification system. Disease activity was assessed using the SLE Disease Activity Index 2000-SLEDAI-2K [20] at the aforementioned time-points. Damage accrual was assessed by the SLICC/ACR Damage Index Score (SDI) [21] (Supplementary Data S1). We also recorded initial and maintenance treatment regimens, time of immunosuppressive and glucocorticoid tapering and discontinuation, as well as the prevalence and time of renal and extrarenal flares at any time over the entire follow-up.

Patients were divided into two subgroups based on their diagnosis period: 1992–2010 (first period) and 2011–2021 (second period).

### Definitions

Renal response and flares were defined according to the 2012 EULAR/ERA-EDTA guidelines [15] and the 2021 and 2024 KDIGO recommendations [16, 17]. The definition of flares is presented in Supplementary Data S2. The majority of renal flares in our cohort (84%) were histologically confirmed by a renal biopsy.

CRR was defined as proteinuria <500 mg/24-h and normal or stable GFR (within 10% of the baseline values). Sustained CRR (sCRR) was defined as a CRR lasting for at least 12 consecutive months. Time to achieve sCRR was calculated from the time of LN diagnosis, and the duration of sCRR from the time of CRR achievement until a renal flare, progression to ESKD, death or EFU. Persistent use of hydroxychloroquine (HCQ) was defined as treatment for at least 2/3 of the follow-up time (from LN diagnosis until sCRR achievement or until the EFU). A ≥ 30% reduction in eGFR at the last follow-up visit compared with the baseline levels was considered as severe kidney function decline. A composite adverse outcome of progression to ESKD or death was evaluated, as there were a few cases of each event in our cohort.

Further details on definitions are presented in Supplementary Data S2.

### Statistical analysis

Continuous variables are expressed as median and interquartile range (IQR) due to their non-normal distribution, while categorical variables are presented as frequencies and percentages. The Mann–Whitney *U* test was used to compare continuous variables between groups. The χ^2^ test was used to compare categorical variables when their assumptions were met; otherwise, Fisher’s exact test was employed. Linear, logistic and Cox proportional hazards regression models were applied to investigate the predictors of sCRR achievement and its impact on the risk of flares, severe kidney function decline, progression to the composite adverse outcome of ESKD/death and organ damage. Kaplan–Meier survival curves were also generated. Significant variables from the univariate analyses were included in the multivariate models, which were refined using stepwise backward selection. Effect measures [β-coefficients (unstandardized and standardized), odds ratios (ORs), hazard ratios (HRs)], *P*-values and 95% confidence intervals (CIs) for the univariate and multivariate models are presented.

To determine which aspect of CRR and sCRR (achievement, timing and duration) better predicts long-term outcomes, we assessed their clinical significance across all models, using the effect size alongside the overall statistical fit of each model. Further details are presented in Supplementary Data S3.

Among patients with extended follow-up (*n* = 91 patients with ≥100 months, corresponding to the upper two tertiles of follow-up duration in our cohort), we additionally explored the minimum duration of sCRR required to confer a protective effect against adverse long-term outcomes. In this subgroup, we further investigated whether sCRR could be achieved and maintained despite reduced exposure to immunosuppressives or glucocorticoids.

Data analysis was conducted using Stata 18.0 software (StataCorp, 2023, Stata Statistical Software: Release 18, College Station, TX, USA; StataCorp, LLC). Significance was set at α = 0.05; all tests were two-tailed.

## Results

The baseline demographic, clinical, laboratory, histological and treatment characteristics of 142 White patients with LN are shown in Supplementary Table S1. The median follow-up time was 121 (IQR: 90) months.

### Prevalence and duration of sCRR

In total, 118/142 (83%) patients achieved sCRR for ≥12 months (Supplementary Fig. S1). Eighty (56.3%) maintained sCRR for at least 5 years, while 20.4% did so for over a decade. Patients who never achieved sCRR for ≥12 consecutive months had slightly lower eGFR (82.5 *vs* 99 ml/min/1.73 m^2^, *P*-value = 0.09), higher prevalence of proliferative LN (PLN) (83.3% *vs* 68.4%, *P*-value = 0.07) and moderate/severe IF/TA (20.8% *vs* 8.8%, *P*-value = 0.08) at baseline compared with sCRR achievers, although these differences did not reach statistical significance. They were also less likely to have received maintenance therapy with mycophenolic acid derivatives (MPA/MMF) (60.8% *vs* 77.1%, *P*-value = 0.06). sCRR achievers had a higher median SLEDAI-2K, lower C4 levels and more frequently positive anti-dsDNA at baseline. All other clinical and laboratory characteristics were comparable between the two groups (Supplementary Table S1). Similar differences between sCRR achievers and non-achievers were observed when groups with different minimum duration of sCRR were compared (e.g. 2, 3, 4 or 5 years) (data not shown).

We studied differences in sCRR achievement and its duration between patients diagnosed in 1992–2010 *vs* the 2011–2021 period. The overall rate of sCRR achievement was comparable (87% *vs* 79.6%, *P*-value = 0.23). Although patients diagnosed in the latter period had a shorter median duration of sCRR (6.1 *vs* 8.9 years, *P*-value = 0.001) (explained by their shorter duration of follow-up), they had spent a greater proportion of their follow-up time on sCRR (89.5% *vs* 78.7%, *P*-value* = *0.014) (Supplementary Table S2). Additional differences included older age at diagnosis (median 36 *vs* 29, *P*-value = 0.004) and significantly greater use of HCQ both at diagnosis (72.1% *vs* 10.9%) and throughout follow-up (71.2% *vs* 7.4%, both *P*-values ≤ 0.001) in the group of patients diagnosed between 2011 and 2021. Νo other clinical/laboratory characteristic or immunosuppressive treatment (initial and maintenance) was significantly different between the two groups.

### Predictors of time to achieve sCRR

**Figure 1.**
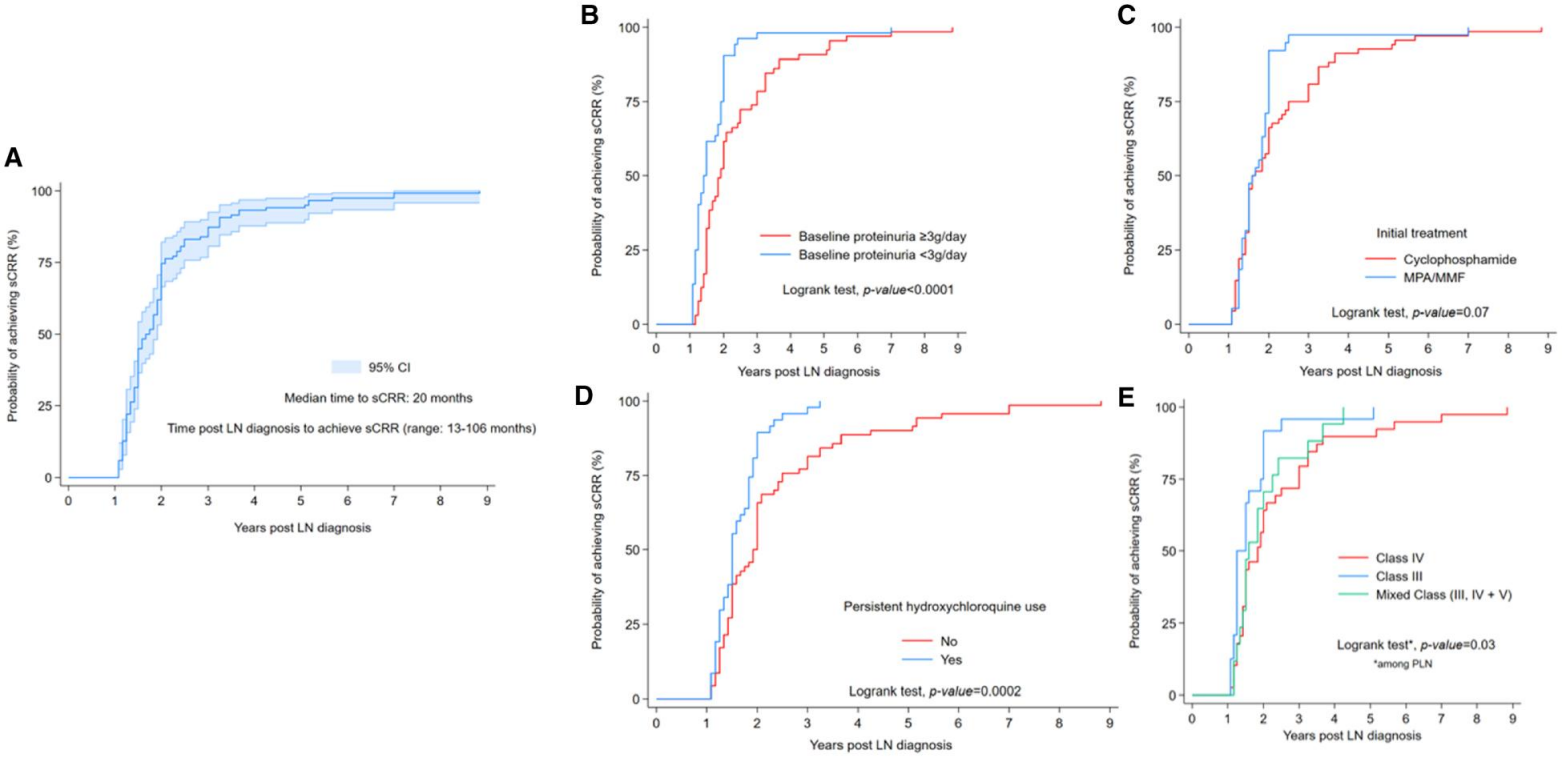
Probability of achieving sustained CRR for ≥12 months during follow-up. **(A)** In the total total cohort, and according to **(B) **baseline levels of proteinuria, **(C)** initial treatment, **(D)** persistent use of hydroxychloroquine (≥2/3 of follow-up from LN diagnosis until sCRR achievement) and **(E)** LN class (among patients with proliferative LN) CI: confidence intervals; LN: lupus nephritis; MPA/MMF: mycophenolic acid/mycophenolate mofetil; PLN: proliferative lupus nephritis; sCRR: sustained complete renal response.

The median time to achieve sCRR for ≥12 months was 20 months post-LN diagnosis (range 13–106 months, Fig. 1A). Most patients (94%) achieved sCRR within five years of LN diagnosis. The most important determinants of shorter time to achieve sCRR for ≥12 months were the use of HCQ at LN diagnosis (HR: 1.65, *P*-value < 0.01), persistent HCQ use (≥2/3 of the follow-up time from LN diagnosis until sCRR achievement) (HR: 2.1, *P*-value < 0.01), non-nephrotic baseline levels of proteinuria (HR: 2.02, *P*-value < 0.001) and initial treatment with MPA/MMF (HR: 1.41, *P*-value = 0.08) (Fig. 1B–D).

In multivariate analysis, persistent use of HCQ (adjusted HR: 1.86, *P*-value* = *0.004) and non-nephrotic baseline proteinuria (adjusted HR: 1.71, *P*-value = 0.016) were independently associated with a shorter time to achieve sCRR. HCQ use at LN diagnosis was not statistically significant in the multivariate analysis (adjusted HR: 1.22, *P*-value > 0.10). The results were consistent in the subgroup analysis of patients with PLN classes; non-nephrotic baseline proteinuria (adjusted HR: 2.30, *P*-value = 0.01) and persistent HCQ use were found to be statistically significant (data not shown). Moreover, patients with class III (HR: 1.89, *P*-value = 0.018) were more likely to achieve sCRR earlier than those with class IV (Fig. 1E).

Of note, incident renal flares significantly decreased the likelihood of ever achieving sCRR; among the 24 patients who never achieved sCRR, 7/24 (29%) experienced a renal flare after renal response. In contrast, only 9/118 (8%) of those who ultimately achieved sCRR experienced a renal flare before sCRR (OR: 0.20, *P*-value = 0.005).

### Renal and extrarenal flares post sCRR

**Figure 2.**
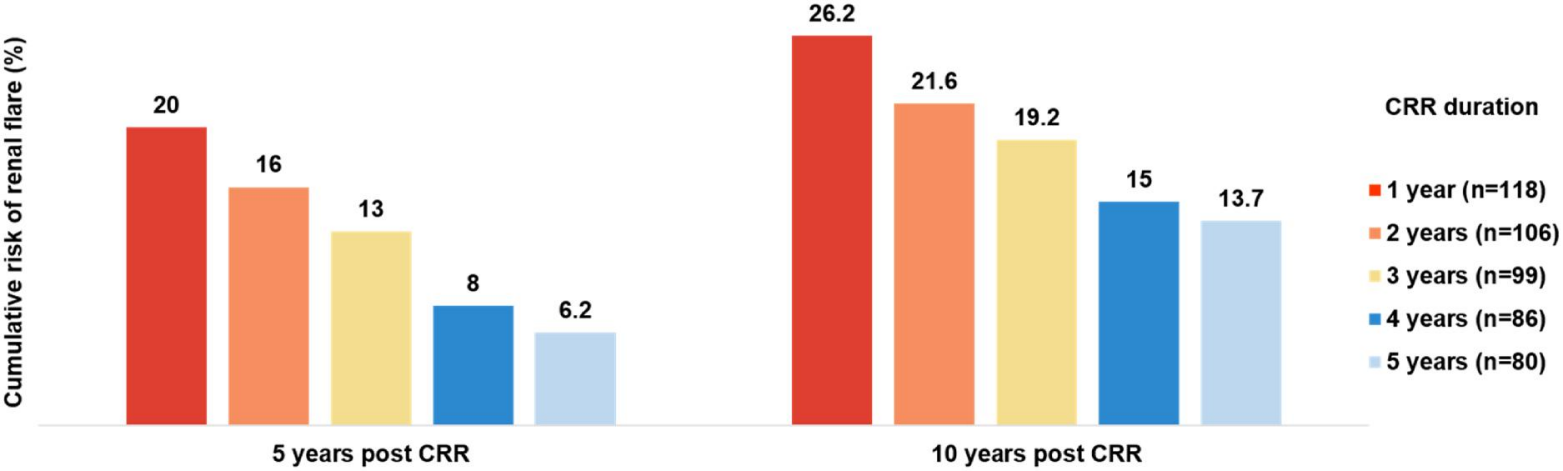
Impact of sustained complete renal response (sCRR) duration on 5- and 10-year cumulative risk of renal flares. Cox proportional hazard models were implemented to estimate the cumulative 5- and 10-year risk of renal flares according to the duration of sustained CRR (1, 2, 3, 4, or 5 years). CRR: complete renal response; n: the number of patients who achieved a minimum of 1, 2, 3, 4, 5 years of sCRR

Forty-four patients (37.3%) experienced flares after attaining sCRR for ≥12 months, in a median time of 43.5 (IQR: 43.5) months post-CRR. Among those, 32 patients (27.1%) experienced renal flares [median time 44 (ΙQR: 46) months post-CRR] and 16 patients (14%) had extrarenal flares [median time 33 (IQR: 42) months post-CRR]. Four patients experienced both renal and extrarenal flares. The estimated cumulative risk for renal flares at 5 and 10 years post-sCRR (sCRR defined as CRR ≥ 12 months) was 20% and 26.2%, respectively. The risk steadily declined with increasing duration of sCRR. Specifically, for patients who maintained sCRR for 2, 3, 4 and 5 years, the 5-year cumulative risk of renal flares was 16%, 13%, 8% and 6.2%, respectively. Similarly, the corresponding 10-year risks were 21.6%, 19.2%, 15% and 13.7% (Fig. 2).

### Achievement and duration of sCRR and risk for severe kidney function decline

Achieving sCRR for ≥12 months, compared with non-achieving sCRR, was protective against severe kidney function decline (OR: 0.125, *P*-value < 0.001). In comparison, CRR (not sustained) was also associated with a reduced risk for severe eGFR decline (OR: 0.20, *P*-value = 0.02), but sCRR provided a stronger effect (Supplementary Table S3A). Among patients who achieved sCRR for ≥12 months, the risk for ≥30% eGFR reduction from baseline levels was 2% lower for every additional year spent on sCRR (OR: 0.98, *P*-value = 0.04) (Table 1). A longer time to achieve sCRR was associated with an increased risk for severe kidney function decline (OR: 1.03/month, *P*-value = 0.03), with those who achieved sCRR ≥ 3 years after LN diagnosis having a 4-fold risk for severe decline (OR: 4.01, *P*-value = 0.03) compared with those achieving sCRR within 3 years from diagnosis (Supplementary Table S3A).

**Table 1.** Predictors of severe kidney function decline (≥30% eGFR reduction compared with baseline levels) during follow-up

Variables	Univariate models		Multivariate model^a^	
	OR	95% CIs * P*-value	OR	95% CIs *P*-value
Achievement of sCRR	0.125	0.04, 0.33 ** <0.001**	0.34	0.07, 1.57 0.17
Duration of sCRR (years)	0.98	0.94, 0.98 ** 0.04**	0.81	0.69, 0.96 ** 0.015**
Persistent^b^ HCQ use	0.15	0.04, 0.54 ** 0.004**	0.07	0.02, 0.31 ** <0.001**

a Backward stepwise logistic regression model; dependent variables: achievement and duration of sCRR, baseline eGFR levels, LN class and persistent use of HCQ.

b ≥2/3 of follow-up from LN diagnosis until the end of follow-up.

CIs: confidence intervals; eGFR: estimated glomerular filtration rate using the CKD-EPI formula; HCQ: hydroxychloroquine; OR: odds ratio; sCRR: sustained complete renal response.

Bold font highlights significant results.

When we evaluated the impact of both the achievement and duration of sCRR on severe kidney function decline, considering baseline eGFR levels, LN class and the persistent use of HCQ, all significant in univariate analysis, only the duration of sCRR (adjusted OR: 0.81, *P*-value = 0.015) and persistent HCQ use (adjusted OR: 0.07, *P*-value < 0.001) remained independently associated with preserved kidney function, while sCRR achievement *per se* (adjusted OR 0.34, *P*-value = 0.17) did not (Table 1).

In the subgroup analysis of patients with extended follow-up, sCRR duration of minimum 3 years showed a marginal trend (OR: 0.22, *P*-value = 0.07), while sCRR duration of at least 4 years (adjusted OR: 0.10, *P*-value = 0.005) was significantly associated with lower risk of severe kidney function decline (Table 4), along with persistent HCQ use (adjusted OR: 0.07, *P*-value = 0.02).

### Achievement and duration of sCRR and risk for the composite adverse outcome of ESKD and death

During follow-up, 13 patients progressed to ESKD and 11 died (six of whom had progressed to ESKD). Achievement of CRR was associated with a lower risk of progression to ESKD/death (HR: 0.21, *P*-value < 0.001), while achievement of sCRR for ≥12 months was associated with an even greater reduction in the risk (HR: 0.09, *P*-value < 0.001) for the composite adverse outcome (Table 2 and Supplementary Table S3B). Among patients who achieved sCRR, each additional year in CRR was associated with a 19% reduced risk of the composite adverse outcome.

**Table 2.** Predictors of the composite adverse outcome (end-stage kidney disease and death)

Variables	Univariate models		Multivariate model^a^		Multivariate model 2^b^	
	HR	95% CIs * P*-value	HR	95% CIs * P*-value	HR	95% CIs * P*-value
At LN diagnosis						
eGFR (per ml/min/1.73 m^2^)	0.96	0.95, 0.98 ** <0.001**	0.97	0.96, 0.99 ** 0.004**	0.96	0.95, 0.98 ** 0.002**
IF/TA (moderate/severe *vs* none/mild)	7.45	2.44, 22.7 ** <0.001**	3.59	1.01, 12.7 ** 0.048**	Not selected	
Chronicity index (per unit)	1.50	1.13, 1.99 ** 0.005**	—	—	1.40	1.06, 1.86 ** 0.017**
Achievement of sCRR	0.09	0.03, 0.24 ** <0.001**	Not selected		Not selected	
Duration of sCRR (per year)	0.81	0.66, 0.93 ** 0.03**	0.75	0.63, 0.89 ** 0.001**	0.74	0.58, 0.94 ** 0.014**

a Backward stepwise Cox regression model; dependent variables: achievement and duration of sCRR, baseline eGFR levels, LN class, hypertension and IF/TA.

b Backward stepwise Cox regression model, only among proliferative LN; dependent variables: achievement and duration of sCRR, baseline eGFR levels, hypertension, IF/TA, activity and chronicity indexes.

CIs: confidence intervals; eGFR: estimated glomerular filtration rate using the CKD-EPI formula; HR: hazard ratio; IF/TA: interstitial fibrosis/tubular atrophy; LN: lupus nephritis; sCRR: sustained complete renal response.

Bold font highlights significant results.

Two Cox regression models were built to evaluate the association of sCRR with the composite adverse outcome in the entire cohort (model 1) and in patients with PLN (model 2). Both sCRR achievement and sCRR duration were included, along with other significant clinical confounders. After backward stepwise selection, sCRR duration, rather than achievement alone, remained independently associated with the composite outcome (adjusted HR: 0.75 and 0.74, *P*-values ≤ 0.014, in model 1 and 2, respectively) (Table 2), alongside baseline eGFR and IF/TA in model 1, and baseline eGFR and chronicity index in model 2.

Among patients with ≥100 months follow-up, the increase in sCRR duration was associated with a stepwise decrease in the risk of the composite outcome, but only sCRR ≥ 4 years was statistically significant (HR: 0.10, *P*-value = 0.037, Supplementary Fig. S2). This effect remained significant in the multivariate analysis (adjusted HR: 0.11, *P*-value = 0.043, Table 4).

### Achievement and duration of sCRR and risk for damage accrual

Patients who achieved sCRR ≥ 12 months were less likely to have accrued damage (SDI ≥ 1) at the EFU compared with those who did not (31.9% *vs* 72.2%, *P*-value = 0.002) and had a lower average annual SDI increase (0.09 *vs* 0.15 units/year, *P*-value = 0.08). In univariate analysis, both CRR and sCRR were significantly associated with lower SDI at the EFU, with sCRR showing a stronger effect (β-coef=−1.22 *vs* –0.85, F-statistic = 18.57 *vs* 6.14, R^2^=0.12 *vs* 0.04), and a more pronounced association (Supplementary Table S3C). Higher baseline eGFR levels, initial treatment with MPA/MMF (*vs* CYC), none/mild (*vs* moderate/severe) IF/TA, absence of hypertension and use of HCQ (both at LN diagnosis and persistently until the EFU) were also associated with lower SDI at the EFU. SDI slightly increased for every additional 5 years of follow-up (β-coef = 0.23, *P*-value = 0.01) and accordingly, patients diagnosed in period 2 had lower SDI, compared with those of period 1 (Table 3).

**Table 3.** Predictors of long-term disease damage

Variables	Univariate models		Multivariate model^a^		Standardized β-coef.^c^
	β-coef.	95% CIs * P*-value	β-coef.	95% CIs * P*-value	
Period of LN diagnosis 1992–2010 (period 1)	Reference category				
2011–2021 (period 2)	−0.55	−0.95, −0.13 ** 0.009**	−0.37	−0.90, −0.16 0.17	−0.15
Follow-up duration^b^ (per 5 years)	0.23	0.05, 0.41 ** 0.013**			
At LN diagnosis					
eGFR (per ml/min/1.73 m^2^)	−0.01	−0.02, −0.01 ** <0.001**	−0.007	−0.01, −0.0004 ** 0.036**	−0.21
IF/TA none/mild	Reference category				
moderate/severe	0.94	0.26, 1.62 ** 0.007**	0.52	−0.14, 1.18 0.12	0.13
HCQ use At LN diagnosis	−0.44	−0.87, −0.02 ** 0.04**	—	—	—
Persistent^d^	−0.55	−0.96, −0.14 ** 0.009**	−0.26	−0.76, 0.07 0.08	−0.10
Initial treatment CYC	Reference category				
MPA/MMF	−0.45	−0.91, 0.002 0.051	−0.21	−0.77, 0.19 0.19	−0.15
Hypertension	−0.42	−0.91, 0.07 0.09	0.06	−0.43, 0.55 0.81	0.02
Achievement of sCRR	−1.22	−1.79, −0.65 ** <0.001**	−1.17	−1.71, −0.63 ** <0.001**	−0.35

a Hydroxychloroquine at baseline and persistent use showed a strong collinearity, and only the latter was used in the final multivariate model.

b Follow-up duration and the period of LN diagnosis showed a strong collinearity, and only the latter was used in the final multivariate model.

c Standardized coefficients beta of the multivariate models.

d ≥2/3 of follow-up from LN diagnosis until the end of follow-up.

β-coef.: β-coefficient; CIs: confidence intervals; CYC: cyclophosphamide; eGFR: estimated glomerular filtration rate using the CKD-EPI formula; HCQ: hydroxychloroquine; IF/TA: interstitial fibrosis/tubular atrophy; LN: lupus nephritis; MPA/MMF: mycophenolic acid/mycophenolate mofetil; sCRR: sustained complete renal response.

In the multivariate model, the achievement of sCRR (adjusted β-coef=−1.17, *P*-value < 0.001) was the strongest predictor of reduced damage accrual. Higher baseline eGFR (adjusted β-coef=−0.007, *P*-value = 0.036) was also independently associated with lower SDI. Persistent HCQ was associated with reduced damage accrual, but it did not reach statistical significance (adjusted β-coef=−0.26, *P*-value = 0.08) (Table 3).

For patients with ≥100 months follow-up, the average annual SDI increase remained markedly lower for patients with sCRR ≥ 12 months (0.13 *vs* 0.57 units/year, *P*-value* = *0.006), while achieving sCRR for at least 4 years was significantly associated with lower SDI at the EFU (β-coef=–0.63, *P*-value = 0.03) and this association remained significant in multivariate analysis (β-coef=−0.81, *P*-value = 0.012) (Table 4). A marginal association was also found with the 3-year sCRR duration (β-coef=−0.68, *P*-value = 0.06).

**Table 4.** Effect of sustained complete renal response lasting ≥4 years (*vs* <4 years) on long-term outcomes in lupus nephritis

Models^a^	Severe (≥30% eGFR reduction compared to baseline levels) kidney function decline	Composite unfavorable outcome (ESKD/death)	Damage accrual
Variables	OR 95% CIs (*P*-value)	HR 95% CIs (*P*-value)	β-coef. 95% CIs (*P*-value)
sCRR <4 years	Reference category		
≥4 years	0.10 0.04, 0.81 (**0.005**)	0.11 0.01, 0.98 (**0.043**)	−0.81 −1.23, −0.03 (**0.012**)
Persistent^b^ HCQ use	0.07 0.01, 0.69 (**0.02**)	—	—
eGFR (per ml/min/1.73 m^2^) at LN diagnosis	—	0.95 0.92, 1.02 (0.06)	−0.10 −0.10, −0.003 (**0.005**)

a Backward stepwise regression models; dependent variables: duration of sCRR ≥ 4 years, baseline eGFR levels, LN class and persistent HCQ use.

b ≥2/3 of follow-up from LN diagnosis until the end of follow-up.

β-coef.: β-coefficient; CIs: confidence intervals; eGFR: estimated glomerular filtration rate using the CKD-EPI formula; ESKD: end stage kidney disease; HR: hazard ratio; HCQ: hydroxychloroquine; LN: lupus nephritis; OR: odds ratio; sCRR: sustained complete renal response.

Bold font highlights significant results.

### Immunosuppressive and glucocorticoid treatment status and sCRR

We further examined the association between sCRR and tapering or discontinuation of immunosuppressive and glucocorticoid treatment. The attempt to taper immunosuppressives was more common among sCRR achievers than non-achievers (90% *vs* 45%, *P*-value < 0.0001, Supplementary Table S4A). Patients, in whom tapering was attempted, had been on CRR for a median time of 2.6 years before immunosuppressives’ tapering initiation and 73.3% of them were able to maintain CRR for a median of 5.5 (IQR: 5.8) years after tapering.

Although immunosuppressive discontinuation was more common in those on sCRR, the association was not statistically significant (*P*-value = 0.12), unless the sCRR had lasted at least 4 years (*P*-value = 0.024). Patients achieved complete immunosuppressive discontinuation after being on sCRR for 4.6 years, with 74% of them maintaining sCRR for a median of 4.5 (IQR: 6) years post-immunosuppressive withdrawal (Supplementary Table S4B). Regarding glucocorticoids, 102 patients were able to discontinue glucocorticoids after a median time of 2 years in CRR.

In the subgroup of patients with ≥100 months follow-up, we further investigated whether sCRR ≥ 4 years could be achieved and maintained despite a reduced exposure to immunosuppressives or glucocorticoids. At the time of achieving sCRR for 4 years, 26% (21/82) of patients were on a full dose of immunosuppressives, 44% were on tapering and 30% (25/82) had discontinued immunosuppressives (Supplementary Fig. S3). Additionally, 78% of patients had discontinued glucocorticoid treatment by this time. Fifteen patients (18%) experienced a renal flare after achieving sCRR for 4 years, with a median time to flare of 25 (IQR: 27) months.

## Discussion

In this 10-year follow-up study, we assessed the feasibility and optimal duration of sCRR in inception cohort patients with biopsy-proven LN. We found that persistent hydroxychloroquine use and absence of flares were associated with earlier CRR achievement, while sCRR for ≥4 years protected against eGFR decline ≥30% from baseline levels, ESKD or death, and damage accrual.

In this cohort of White individuals with LN, 83% of patients achieved sCRR within a median time of 20 months post-LN diagnosis, with over 20% of them maintaining it for more than a decade. Persistent HCQ use significantly increased the likelihood of achieving remission earlier, both in the entire cohort and in the subgroup of patients with PLN. This observation adds to the well-established benefits of HCQ in preventing renal and extrarenal flares of the disease [22–26]. Renal flares reduced the likelihood of ever achieving sCRR, which likely reflects the cumulative nephron loss and structural injury associated with each flare episode that progressively limit renal recovery capacity [27–29] and highlights the importance of early disease control [30–32]. Notably, patients with class III LN were more likely to achieve sCRR earlier than those with class IV LN, indicating the impact of class IV on maintained renal response [33] in addition to its well-known effect on renal survival [3].

Renal flares pose a challenge in the management of LN due to their frequency (25–66% in various cohorts), and association with progressive kidney disease [30, 34, 35]. Although 83% of our study population achieved sCRR, disease flares remained a concern, with 37.3% of patients experiencing a flare at a median time of 43.5 months post-CRR. However, we demonstrated that each additional year of sustainedly attained CRR was associated with reduced risk of renal flare. Gatto *et al.* showed that prolonged clinical remission defined as GFR > 60 ml/min/1.73 m^2^ lasting >1 year and clinical SLEDAI = 0 significantly decreased the risk of SLE flares [12].

In our study, we aimed to compare the impact of achievement, timing and duration of CRR and sCRR on long-term outcomes. Through a thorough analysis encompassing the three major study outcomes, we consistently observed that the achievement of sCRR was the strongest and most consistent predictor of favorable prognosis, with every added year on CRR offering additional benefit. Specifically, we found that each additional year on sCRR reduced the risk of severe eGFR decline by 19%, and the risk of ESKD or death by 25%. Our findings regarding the protective role of sCRR align with our previous data on disease modification in LN. These data showed that failure to fulfill the criteria for flare, proteinuria and eGFR levels at each of the time frames was associated with poorer long-term renal outcomes [36]. The results of the current study emphasize the importance of maintaining long-term sCRR as a key therapeutic goal in LN.

Achievement of sCRR and its duration protected against damage accrual, as indicated by lower SDI increase during follow-up and lower scores at the EFU. Few previous studies have examined the duration of sustained remission as potential predictor of renal outcomes and disease damage. However, definitions varied among these studies with some reporting on renal response and others on clinical remission, including renal and extrarenal parameters incorporated by the SLEDAI score [11–13, 37]. In addition, some studies used pre-defined cut-off timepoints (e.g. >5 *vs* <5 years) [13] and some compared patients with sustained remission to those who never achieved it [37]. We observed lower odds of severe kidney function decline, composite outcome of ESKD or death and damage in patients with a minimum of 4 years sCRR and a marginal association with a minimum of 3 years sCRR.

An interesting finding of our study is that although the overall rate of sCRR achievement was comparable between the two study periods, patients in the second period had spent a higher proportion of their follow-up in CRR. This could be explained by evolving treatment practices, particularly the markedly greater persistent use of HCQ, which emerged as a significant predictor of shorter time to sCRR and preservation of kidney function, supporting its continued use as a key component of LN therapy [25].

Novel agents such as belimumab, voclosporin and obinutuzumab have shown, in large clinical trials, to induce earlier and higher rates of CRR when added to standard of care [29, 38–40]. In our cohort, these agents were not used, as patients were managed before their regulatory approval (the last patient was diagnosed in 2021). However, our findings demonstrate that sCRR can be achieved with conventional therapies, further supporting the use of these novel agents to enhance early response and improve long-term outcomes.

While previous studies have examined the effect of sustained remission on long-term outcomes in LN patients, we further demonstrated that after 4 years of sCRR, patients could taper or discontinue immunosuppressives with a high likelihood of maintaining remission. Specifically, at the time a 4-year sCRR was achieved, we found that 78% of patients had discontinued glucocorticoids, while 74% had either tapered or discontinued immunosuppressives. Subsequent renal flares occurred in only 18% of patients after 4 years of sCRR, indicating long-term sustained remission may allow for successful tapering of immunosuppression.

This study has several strengths. Firstly, it is based on well-characterized inception cohort LN patients from two academic centres with consistent long-term (median 10-year) follow-up data. Secondly, we adopted the definition of CRR recommended by EULAR and KDIGO guidelines [15–17], enhancing the clinical applicability of our findings. This standardized definition makes our results more relevant to practicing clinicians, providing a framework for assessing renal response in routine care. Although the observational design of the study does not allow us to establish a causal link between sCRR and favorable long-term outcomes, the inclusion of exclusively inception cohort patients in the study and the frequent recording of multiple clinical/laboratory/treatment characteristics, as well as adjustments for these characteristics, the treatment exposure, and a possible effect of the diagnostic period on outcomes, are among the strengths of the study. A potential limitation of the current study is the lack of per-protocol biopsies. These biopsies could provide additional insights into histopathological remission of the disease, which does not always align with the clinical response [41–43]. Additionally, the predominance of White participants and the study’s setting within a public healthcare system with free medical care may limit the generalizability of our findings to more diverse ethnic groups and different healthcare environments.

In conclusion, achieving sCRR is a clinically meaningful goal in patients with LN, significantly improving long-term renal and patient survival and reducing damage accrual. In our study, persistent HCQ use, non-nephrotic baseline proteinuria and class III *vs* class IV appeared to significantly impact the time to sCRR. Maintaining sCRR for at least four years is inversely associated with long-term risk of severe kidney function decline, ESKD/deaths and damage accrual.

## Supplementary Material

keaf677_Supplementary_Data

## Data Availability

Data supporting the study’s findings are available from the corresponding author upon reasonable request.

## Notes

*Patient and public involvement*: Patients and/or the public were not involved in the design, conduct, reporting or dissemination of this research.
