# Supplementary material for: Predictors and long-term impact of sustained complete renal response in lupus nephritis patients over a median 10-year follow-up
Source: Rheumatology (Oxford). 2025 Dec 13;65(3):keaf677. doi: 10.1093/rheumatology/keaf677 (PMC13017678; doi:10.1093/rheumatology/keaf677)
Supplement: keaf677_Supplementary_Data [file keaf677_supplementary_data.docx]

**Supplementary Materials**

**Supplementary Data S1 – Data Collection**

*Damage accrual assessment*

Damage accrual was evaluated using the SLICC/ACR Damage Index (SDI) score (1). At the end of follow-up (EFU), 62.6% of patients had no damage accrual (SDI=0). For the remaining patients, we reviewed the medical charts between years 3 and 6 of follow-up (the minimum follow-up duration in our cohort was 3 years) which provided an additional informative time point for assessing early SDI changes. To ensure consistency of recorded damage progression, clinical charts 12 months before and 12 and 24 months after this window were also reviewed.

**Supplementary Data S2 – Definitions**

- Flares were defined as follows: nephritic flares as a reproducible ≥10% decrease in GFR accompanied by an active urine sediment with ≥10 red blood cells per high-power field, irrespective of changes in proteinuria. Proteinuric flares were defined as a reproducible doubling of proteinuria to >1000 mg/24h after complete response or to >2000 mg/24h after partial response (2).
- Persistent use of hydroxychloroquine (HCQ) was defined as treatment for at least 2/3 of the follow-up time from lupus nephritis (LN) diagnosis. In specific, persistent use of HCQ was evaluated in two different contexts in our study:

1. from LN diagnosis until the achievement of sustained complete renal response (sCRR) in the analysis of the predictors of time to achieve sCRR and,
2. from LN diagnosis until the last follow-up visit in the models for long-term outcomes (≥30% eGFR decline, ESKD/death, and damage), because these analysesincluded patients who never achieved sCRR.

- End of follow-up (EFU) was defined as the time of end-stage kidney disease (ESKD), death, or the date of the last recorded clinical visit, whichever occurred first. For patients without an event, the last clinical assessment was used as the censoring point. This approach ensured consistent determination of follow-up duration across all analyses, including renal outcomes (eGFR decline, ESKD/death) and damage accrual.

**Supplementary Data S3 - Statistical analysis**

*Comparative evaluation of achievement, timing and duration of CRR and sCRR across long-term outcomes*

To compare the predictive strength of CRR and sCRR across all major long-term outcomes (severe kidney function decline, progression to ESKD/death, and organ damage), multiple regression models were developed according to the outcome type: logistic regression for severe kidney function decline, Cox proportional hazards regression for progression to ESKD/death and linear regression for damage. For each model, effect measures appropriate to the outcome were reported, including β-coefficients (unstandardized and standardized), odds ratios (ORs), and hazard ratios (HRs) with corresponding p-values. To evaluate the robustness and explanatory capacity of each model, several indicators of statistical fit were calculated: the Likelihood Ratio (LR) chi-square and McFadden’s Pseudo R² for models on severe kidney function decline, the LR chi-square and Harrell’s C index for models predicting the composite outcome of ESKD/death, and the F-statistic and R² for models on organ damage (SDI). This comparative framework enabled a quantitative assessment of both the magnitude of effect (through effect sizes) and the predictive performance (through model fit) for CRR and sCRR, the time to achieve them and their duration, in relation to the long-term outcomes.

*Models on severe kidney function decline*

Severe kidney function decline [≥30% reduction in eGFR from baseline until the end of follow-up (EFU)] was assessed at the end of follow-up (EFU) to reflect a persistent and established loss of renal function, rather than a transient eGFR fluctuation. To confirm that these declines represented established loss of kidney function, only patients with sustained eGFR decline for at least 3 months in the end of follow-up were included as events. For this reason, logistic rather than Cox regression models were used to address this long-term outcome.

*Handling of flares in the statistical models*

1. For the analysis on the overall probability of ever achieving sCRR, all patients were included in the model, and the occurrence or not of a renal flare was analysed as a covariate in the model. For patients who achieved sCRR, only the flares that preceded sCRR were assessed.
2. For the analysis on the post-sCRR risk for flares, patients were censored at the time of the first flare following sCRR achievement, or, if no flare occurred, at the end of follow-up (EFU). Cox proportional hazard models were applied to estimate the flare risk over time and to assess the cumulative probability of renal flares at 5 and 10 years according to different sCRR durations (1, 2, 3, 4 and 5 years of sCRR).

**Supplementary Table S1.** Baseline demographic, clinical, laboratory and histological characteristics and immunosuppressive treatment regimens in the total cohort and among achievers and non-achievers of sustained complete renal response (sCRR).

| **Characteristics** | **Total  (n=142)** | **Achievers of sCRR for ≥12 months**  **(n=118)** | **Non-achievers of sCRR**  **(n=24)** | **p-value** |
| --- | --- | --- | --- | --- |
|  | **Median [IQR], N (%)** | | |  |
| Period of LN diagnosis   - 1992-2010 (1^st^) | 69 (48.6) | 60 (50.9) | 9 (37.5) | 0.26 |
| - 2011-2021 (2^nd^) | 73(51.4) | 58 (49.1) | 15 (62.5) |  |
| - ***At LN diagnosis*** | | | | |
| Age (years) | 33 [19] | 33 [19] | 31.5 [21.5] | 0.99 |
| LN as first manifestation of SLE | 74 (52) | 61 (51.7) | 13 (54.2) | 0.82 |
| Duration of SLE prior to LN (years)* | 5 [8] | 5 [8] | 2 [10] | 0.53 |
| Sex   - Females | 116 (81.7) | 96 (81.4) | 20 (83.3) | 0.82 |
| - Males | 26 (18.3) | 22 (18.6) | 4 (16.7) |  |
| Hypertension | 33 (23.7) | 26 (22.4) | 7 (30.4) | 0.41 |
| eGFR (ml/min/1.73 m^2^) | 96 [46] | 99 [42] | 82.5 [80.5] | 0.09 |
| - ≥60 ml/min/1.73 m^2^ | 113 (80.1) | 97 (83) | 16 (66.7) | **0.013** |
| - 31-59 ml/min/1.73 m^2^ | 15 (10.6) | 13 (11) | 2 (8.3) |  |
| - ≤30 ml/min/1.73 m^2^ | 13 (9.2) | 7 (6) | 6 (25) |  |
| Urine protein excretion (g/24h) | 3 [4] | 3 [4] | 4 [4] | 0.13 |
| - >3 g/24h | 81 (57.4) | 65 (55.6) | 16 (66.6) | 0.25 |
| - 1-3 g/24h | 17 (12.1) | 13 (11.1) | 4 (16.7) |  |
| - <1 g/24h | 43 (30.5) | 39 (33.3) | 4 (16.7) |  |
| SLEDAI-2K | 12 [6] | 12 [4] | 9 [6] | **0.001** |
| Low C3 (≤80 mg/dL) | 82 (67.8) | 72 (69.2) | 10 (58.8) | 0.39 |
| Low C4 (≤9 mg/dL) | 78 (64.5) | 71 (68.3) | 7 (41.2) | **0.03** |
| Positive anti-dsDNA (≥7 mg/dL) | 96 (82.8) | 88 (86.3) | 8 (57.1) | **0.007** |
| Positive urine sediment | 121 (86.4) | 103 (88) | 18 (78.3) | 0.21 |
| LN class   - Membranous | 42 (29.6) | 37 (31.6) | 4 (16.7) | 0.07 |
| - Proliferative (III, IV ± V) | 100 (70.4) | 80 (68.4) | 20 (83.3) |  |
| - III / IV / III, IV + V | 28 (20) / 47 (33.3) / 25 (17.6) | 24 (30) / 39 (48.8) / 17 (21.2) | 4 (20) / 8 (40) / 8 (40) | 0.27 |
| Number of crescents | 0 [3] | 0 [3] | 0 [4] | 0.78 |
| Glomerulosclerosis** (%) | 7.7 [18.7] | 7.6 [16] | 11.5 [30] | 0.18 |
| Interstitial fibrosis/tubular atrophy***   - none/mild | 123 (89.1) | 104 (91.2) | 19 (79.2) | 0.08 |
| - moderate/severe | 15 (10.9) | 10 (8.8) | 5 (20.8) |  |
| Activity Index**** | 10 [6] | 10 [5] | 11 [7] | 0.48 |
| Chronicity Index**** | 2 [2] | 2 [2] | 2 [4] | 0.97 |
| Initial treatment   - CYC | 81 (63.3) | 68 (64.2) | 13 (59.1) | 0.65 |
| - MPA/MMF | 47 (36.7) | 38 (35.8) | 9 (40.9) |  |
| Maintenance treatment   - MPA/MMF | 105 (74.5) | 91 (77.1) | 14 (60.8) | 0.06 |
| - Other^#^ | 20 (14.5) | 17 (14.4) | 3 (13) |  |
| - None | 16 (11) | 10 (8.5) | 6 (26) |  |
| HCQ use | 49 (35) | 41 (36) | 8 (33) |  |

*: among non-incident LN patients, **: percentage of sclerosed glomeruli among the total number of glomeruli, ***: percentage of the renal cortex involved by interstitial fibrosis and tubular atrophy, ****: only for proliferative LN, #: azathioprine and cyclosporine

anti-dsDNA: anti-double stranded-DNA antibodies, CYC: cyclophosphamide, eGFR: estimated glomerular filtration rate using the CKD-EPI formula, HCQ: hydroxychloroquine, IQR: interquartile range, LN: lupus nephritis, MPA/MMF: mycophenolic acid/mycophenolate mofetil, N/n: number, sCRR: sustained complete renal response, SLE: systemic lupus erythematosus, SLEDAI-2K: systemic lupus erythematosus disease activity index

**Supplementary Table S2.** Baseline characteristics, immunosuppressive treatment and achievement of sustained complete renal response (sCRR) in the 1^st^ (1992-2010) and 2^nd^ (2011-2021) period.

| **Characteristics** | **1^st^ period (1992-2010)**  **(n=69)** | **2^nd^ period (2011-2021)**  **(n=73)** | **p-value** |
| --- | --- | --- | --- |
|  | **Median [IQR], N (%)** | |  |
| Age at LN diagnosis (years) | 29 [13] | 36 [22] | **0.004** |
| Sex (females) | 55 (79.6) | 61 (83.6) | 0.55 |
| LN class (proliferative) | 52 (75.3) | 48 (65.8) | 0.20 |
| Initial treatment   - CYC | 44 (67.7) | 37 (58.7) | 0.29 |
| - MPA/MMF | 21 (32.3) | 26 (41.3) |  |
| Maintenance treatment   - MPA/MMF | 51 (75) | 54 (74) | 0.94 |
| - Other^#^ | 9 (13.2) | 11 (15) |  |
| - None | 8 (11.8) | 8 (11) |  |
| HCQ use   - at baseline | 7 (10.9) | 49 (72.1) | **<0.001** |
| - persistent* | 5 (7.4) | 52 (71.2) | **<0.001** |
| sCRR achievement | 60 (87) | 58(79.6) | 0.23 |
| sCRR duration (years) | 8.9 [8.4] | 6.1 [4.4] | **<0.001** |
| Follow-up (years) | 14.3 [6.3] | 7.1 [4.4] | **<0.001** |
| Percentage of follow-up spent on sCRR | 78.7 [61] | 89.5 [27] | **0.014** |

*: ≥2/3 of follow-up from LN diagnosis until the end of follow-up, #: azathioprine and cyclosporine

CYC: cyclophosphamide, HCQ: hydroxychloroquine, IF/TA: interstitial fibrosis/tubular atrophy, IQR: interquartile range, LN: lupus nephritis, MPA/MMF: mycophenolic acid/mycophenolate mofetil, N/n: number, sCRR: sustained complete renal response

**Supplementary Table S3.** Association of achievement, timing, and duration of complete and sustained complete renal response (CRR/sCRR) with:

1. **severe kidney function decline** (≥30% eGFR reduction compared to baseline levels) during follow-up.

| **Variables** | **OR** | **p-value** | **LR χ^2^** | **Pseudo R^2^** |
| --- | --- | --- | --- | --- |
| Achievement of CRR | 0.20 | 0.02 | 14.2 | 0.10 |
| Time to CRR (per month) | 1.01 | 0.36 | 0.79 | 0.006 |
| Achievement of sCRR | 0.125 | <0.001 | 16.88 | 0.12 |
| Time to sCRR (per month) | 1.03 | 0.03 | 4.29 | 0.05 |
| - ≥3 years post LN diagnosis | 4.01 | 0.03 | 4.31 | 0.05 |
| Duration of sCRR (per year) | 0.98 | 0.04 | 17.3 | 0.13 |

CRR: complete renal response, LN: lupus nephritis, LR χ2: Likelihood Ratio chi-square test, OR: odds ratio, Pseudo R2: McFadden’s Pseudo R2 test, sCRR: sustained complete renal response

1. **the risk for the composite adverse outcome** (end-stage kidney disease and death).

| **Variables** | **HR** | **p-value** | **LR χ^2^** | **Harrell’s C** |
| --- | --- | --- | --- | --- |
| Achievement of CRR | 0.21 | 0.001 | 16.14 | 0.75 |
| Time to CRR (per month) | 0.99 | 0.92 | 0.01 | 0.67 |
| Achievement of sCRR | 0.09 | <0.001 | 21.42 | 0.79 |
| Time to sCRR (per month) | 1.01 | 0.63 | 0.20 | 0.57 |
| - ≥3 years post LN diagnosis | 2.65 | 0.07 | 1.5 | 0.55 |
| Duration of sCRR (per year) | 0.81 | 0.03 | 23.61 | 0.85 |

CRR: complete renal response, HR: hazard ratio, LN: lupus nephritis, LR χ2: Likelihood Ratio chi-square test, sCRR: sustained complete renal response

1. **the risk for long-term disease damage.**

| **Variables** | **β-coef** | **Standardized β-coef** | **p-value** | **F-statistic** | **R^2^** |
| --- | --- | --- | --- | --- | --- |
| Achievement of CRR | -0.85 | -0.20 | 0.02 | 6.14 | 0.04 |
| Time to CRR (per month) | 0.01 | 0.12 | 0.17 | 1.87 | 0.01 |
| Achievement of sCRR | -1.22 | -0.35 | <0.001 | 18.57 | 0.12 |
| Time to sCRR (per month) | 0.01 | 0.18 | 0.05 | 3.85 | 0.03 |
| - ≥3 years post LN diagnosis | 0.93 | 0.30 | 0.001 | 12.5 | 0.10 |
| Duration of sCRR (per year) | -0.02 | -0.10 | 0.28 | 1.16 | 0.01 |

β-coef: β-coefficient, CRR: complete renal response, LN: lupus nephritis, sCRR: sustained complete renal response, Standardized β-coef: Standardized β-coefficient

**Supplementary Table S4**. Association between immunosuppressive and glucocorticoid treatment status and achievement of sustained complete renal response (sCRR).

1. **Immunosuppresives tapering** and **discontinuation** among achievers and non-achievers of sustained complete renal response (sCRR).

| **Immunosuppresives treatment strategy** | **Achievers of sCRR for ≥12 months**  **(n=118)** | **Non-achievers of sCRR**  **(n=24)** | **p-value** |
| --- | --- | --- | --- |
|  | **Median [IQR], N (%)** | |  |
| Immunosuppresives tapering attempted | 101 (90) | 10 (45) | **<0.001** |
| Immunosuppresives discontinuation achieved* | 70 (70) | 5 (50) | 0.12 |

*Among patients in whom immunosuppresives tapering was attempted

IQR: interquartile range, N: number, sCRR: sustained complete renal response

1. Duration of sustained complete renal response **before** and **after immunosuppressives tapering** and **discontinuation**.

| **Immunosuppresives treatment strategy** | **IS tapering attempted** | **IS discontinuation achieved** |
| --- | --- | --- |
|  | **Median [IQR]** | |
| Years on CRR before tapering initiation | 2.6 [2.2] | 4.6 [3.5] |
| Years on CRR after tapering initiation | 5.5 [5.8] | 4.5 [6] |

IQR: interquartile range, IS: immunosuppressives

**Supplementary Figure 1.** Prevalence of sustained complete renal response of different duration during follow-up.


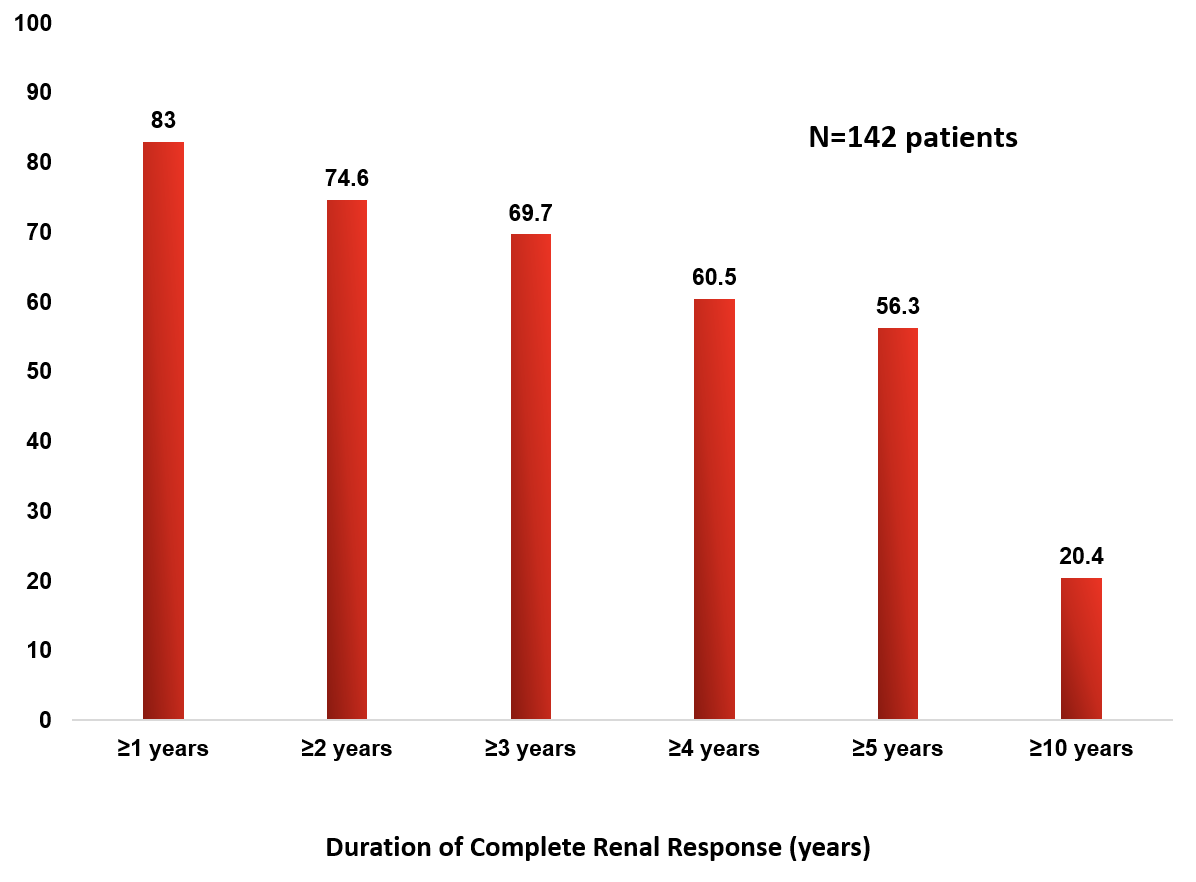


**Alternative text:** Bar chart showing the proportion of patients who achieved sustained complete renal response for at least 1, 2, 3, 4, 5, and 10 years.

**Supplementary Figure 2.** Kaplan–Meier estimates for the survival from the composite adverse outcome [progression to end-stage kidney disease (ESKD) and death], according to the achievement and duration of sCRR.


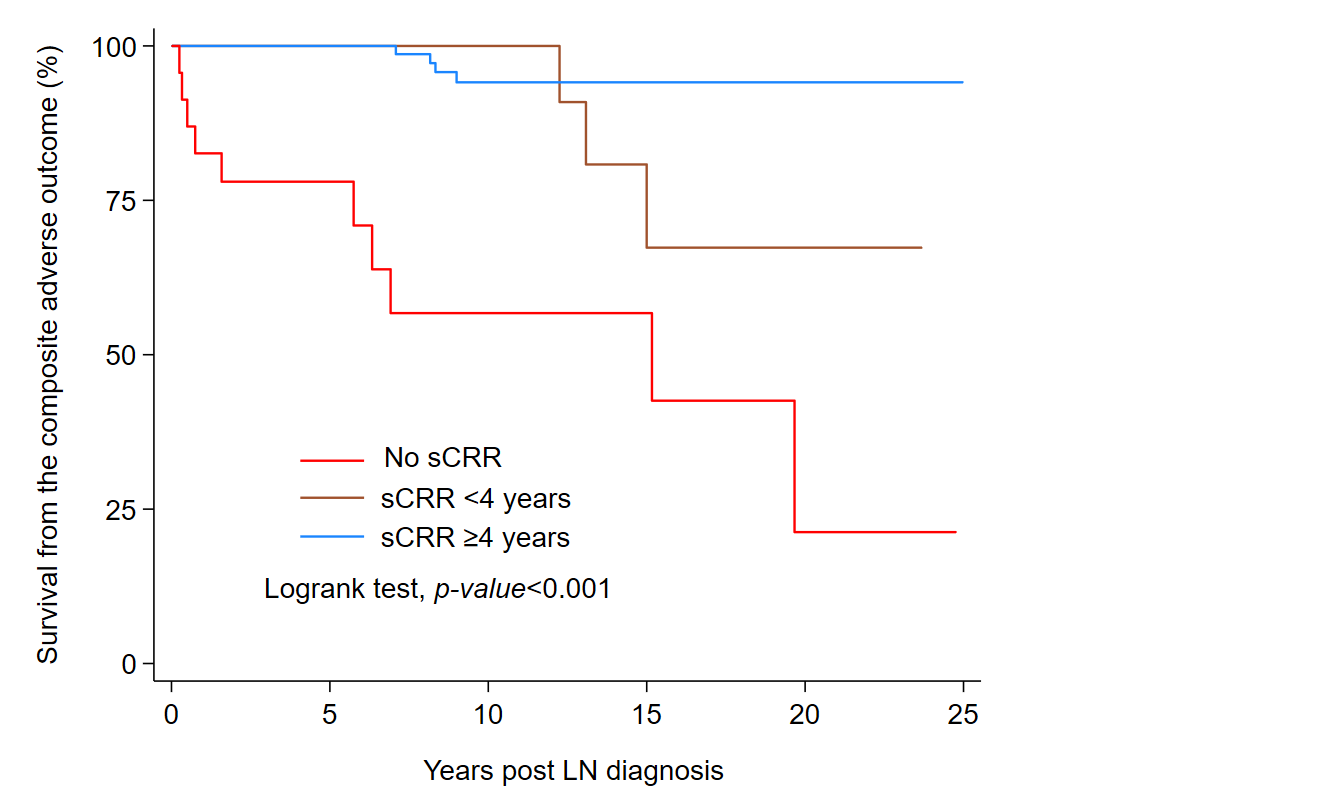


LN: lupus nephritis, sCRR: sustained complete renal response

**Alternative text:** Kaplan–Meier curves comparing the survival from the composite outcome of end-stage kidney disease or death among patients without sustained renal response (sCRR), sCRR lasting <4 years, and sCRR for ≥4 years, showing significantly improved survival with longer sCRR duration.

**Supplementary Figure 3.** Exposure to immunosuppressives by the time sustained complete renal response (sCRR) for ≥4 years was achieved (among patients with ≥100 months of follow-up).

**
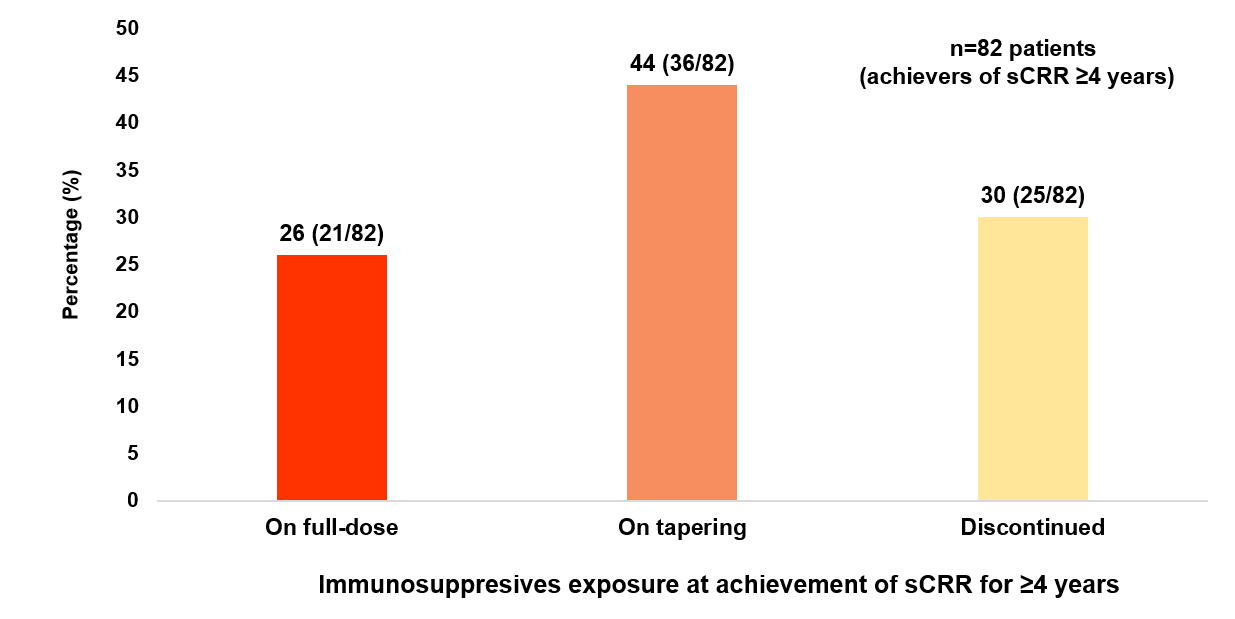
**

n: number, sCRR: sustained complete renal response

**Alternative text:** Bar chart showing immunosuppressive exposure at the time sustained complete renal response for at least four years was achieved, illustrating proportions of patients on full-dose therapy (26%), on tapering (44%), or with treatment fully discontinued (30%).

**References**

1. Gladman DD, Urowitz MB, Goldsmith CH, Fortin P, Ginzler E, Gordon C, et al. The reliability of the Systemic Lupus International Collaborating Clinics/American College of Rheumatology Damage Index in patients with systemic lupus erythematosus. Arthritis Rheum. 1997;40(5):809–13.
2. Bertsias GK, Tektonidou M, Amoura Z, et al. Joint European League Against Rheumatism and European Renal Association-European Dialysis and Transplant Association (EULAR/ERA-EDTA) recommendations for the management of adult and paediatric lupus nephritis. Ann Rheum Dis. 2012;71(11):1771-1782. doi:10.1136/annrheumdis-2012-201940
